# Direct and lost productivity costs associated with avoidable hospital admissions

**DOI:** 10.1186/s12913-020-5071-4

**Published:** 2020-03-13

**Authors:** João Victor Muniz Rocha, Ana Patrícia Marques, Bruno Moita, Rui Santana

**Affiliations:** 1 Universidade Nova de Lisboa Escola Nacional de Saúde Publica Lisboa, Avenida Padre Cruz, 1600-560 Lisboa, Portugal; 2 Universidade Nova de Lisboa Centro de Investigação em Saúde Publica Lisboa, Lisboa, Portugal; 3 Centro Hospitalar Universitário do Algarve, E.P.E Faro, PT. Universidade Nova de Lisboa Escola Nacional de Saúde Publica Lisboa, Rua Leão Penedo, 8000-386 Faro, Portugal

**Keywords:** Hospital admissions, Avoidable admissions, ambulatory care sensitive conditions, Cost analysis

## Abstract

**Background:**

Hospitalizations for ambulatory care sensitive conditions are commonly used to evaluate primary health care performance, as the hospital admission could be avoided if care was timely and adequate. Previous evidence indicates that avoidable hospitalizations carry a substantial direct financial burden in some countries. However, no attention has been given to the economic burden on society they represent. The aim of this study is to estimate the direct and lost productivity costs of avoidable hospital admissions in Portugal.

**Methods:**

Hospitalizations occurring in Portugal in 2015 were analyzed. Avoidable hospitalizations were defined and their associated costs and years of potential life lost were calculated. Direct costs were obtained using official hospitalization prices. For lost productivity, there were estimated costs for absenteeism and premature death. Costs were analyzed by components, by conditions and by variations on estimation parameters.

**Results:**

The total estimated cost associated with avoidable hospital admissions was €250 million (€2515 per hospitalization), corresponding to 6% of the total budget of public hospitals in Portugal. These hospitalizations led to 109,641 years of potential life lost. Bacterial pneumonia, congestive heart failure and urinary tract infection accounted for 77% of the overall costs. Nearly 82% of avoidable hospitalizations were in patients aged 65 years or older, therefore did not account for the lost productivity costs. Nearly 84% of the total cost comes from the direct cost of the hospitalization. Lost productivity costs are estimated to be around €40 million.

**Conclusion:**

The age distribution of avoidable hospitalizations had a significant effect on costs components. Not only did hospital admissions have a substantial direct economic impact, they also imposed a considerable economic burden on society. Substantial financial resources could potentially be saved if the country reduced avoidable hospitalizations.

## Background

Ambulatory care sensitive conditions (ACSCs) are health conditions for which hospital admission could be prevented by timely and adequate ambulatory care [1, 2]. Hospitalizations for ACSCs have been extensively used in health care research and health policies to assess accessibility, quality and performance of the primary health care, as timely and effective primary care could potentially avoid hospitalization [3, 4]. Previous studies in the field have mostly analyzed rates and trends of hospitalizations for ACSCs and the association with different contextual factors; less attention has been given to the economic and social impact these hospitalizations produce.

Direct costs of admissions for ACSCs were estimated in previous studies in Ireland [5], the United Kingdom [6, 7], France [8] and Brazil [9, 10], using the official prices of hospitalizations in each country’s respective national health system. Two studies in Portugal used different ACSC definition methodologies to estimate costs associated with avoidable hospital admissions [11, 12]; the first (in 2007) used the methodology of Agency for Healthcare Research and Quality (AHRQ) [4], while the second (in 2014) used the ACSC list developed by Caminal [13]. These studies found that the direct costs of avoidable hospital admissions amounted to approximately €200 million and €250 million, respectively. Both studies found that around 10% of all hospitalizations in Portugal were potentially avoidable [11, 12], indicating that there is room for improvement in the country regarding ACSCs.

Of the limited number of studies that estimated the costs of avoidable hospitalizations, none of them included estimations of costs for lost productivity, lost wages and premature death. Although the findings of existing studies already indicate that avoidable hospitalizations create a substantial direct financial burden on health expenditures, considering lost productivity costs can further illustrate how much economic pressure such hospitalizations present.

Analysis of the economic burden on society associated with avoidable hospitalizations provides valuable information for planning of health services and allocation of resources. Moreover, as such hospitalizations could have potentially been avoided in the ambulatory care setting; this analysis indicates the potential of saving costs by reducing hospitalizations for ACSCs. The aim of this study was to estimate direct and lost productivity costs of hospitalizations for ACSCs in Portugal. This country provides universal health care, with services financed primarily through tax payments. The primary health care is intended to be the first point of contact of users with the health system.

## Methods

### Data source and sample definition

This study used the hospitalization data base provided by the Portuguese Central Administration of the Health System for the year 2015. A total of 1,000,670 hospitalizations were registered in continental Portugal in 2015. For each hospital admission, the inpatient data used in this study were age, sex, principal and secondary diagnosis (according to ICD-9-CM code), diagnosis-related groups (DRGs), length of stay and reason for end of the hospitalization.

The definition of which hospitalizations were for ACSCs was determined according to the AHRQ methodology, which uses the codes of principal and secondary diagnosis, for different versions of the ICD [4]. This list has a strong theoretical basis for its composition and a well-defined methodology for inclusion of cases and exclusion of some comorbidities [4]. The AHRQ guidelines provide more details in disease coding and inclusion/exclusion criteria [4].

### Cost estimation

The costs associated with hospitalizations for ACSCs were both direct and from a socioeconomic perspective, incurred by lost productivity. The identification of direct costs was done in the total of hospitalizations. The valuation was done according to the number of hospital admissions classified as avoidable. For the monetization of direct costs, official hospital inpatient admission prices were used as proxy for costs. Prices are defined by DRGs and severity of the condition, according to values published by the Ministry of Health [14]. The prices correspond only to the hospital admission and, therefore did not include other pre- and post-hospitalization expenses.

Lost productivity costs were identified for absenteeism and premature death. The valuation was based on to the length of stay and years of potentially productive life lost for avoidable hospitalizations. In order to monetize productivity losses, the human capital approach was applied. In this methodology, the value to society of potentially lost production (either due to absenteeism or premature death) is estimated by market wages [15, 16]. The human capital approach has been used in the literature to estimate lost productivity costs of morbidity and/or mortality for cancer [16–18], asthma [19], *E. coli* infections [20], visual impairment [21] and road traffic accidents [22], to name a few.

For all hospitalizations for ACSCs, the estimated absenteeism cost was estimated as the value of days of productivity lost, calculated as the length of stay multiplied by the daily wage (the monthly mean wage of the region divided by 30 days, as done by previous studies [23–25]), taking into account whether the person was of working age (between 18 and 64 years of age), the probability of the patient being part of the labor force (based on labor force participation) and being employed (based on the unemployment rate).

For avoidable hospitalizations in which the patient died in the hospital, premature deaths are understood as potentially preventable hospitalization-related deaths that occur at working age have a cost of lost productivity that extends to retirement age. The estimated cost of the lost productivity for such premature death was also calculated. In this case, the years of potentially productive life lost were calculated as the difference between the age of the patient and the retirement age (considered 65 years old), and multiplied by the annual mean local wage. Monthly wage, unemployment and labor force participation were specific according to the gender of the patient for both absenteeism and premature death, to reflect the gender differentials in the labor market. These data were obtained at the municipal level from Statistics Portugal (SP).

The methodology used for lost productivity costs only accounts for the working-age population. While it has to be acknowledged that retired people contribute to the production of the country, this was not monetized in this study. For equity, the impact of avoidable hospitalizations in the whole population was taken into consideration. Therefore, this impact was considered illness burdens instead of lost productivity costs. Years of potential life lost (YPLL) were calculated to quantify the burden imposed on society by avoidable hospital admissions. YPLL were calculated to represent the non-financial impact these hospitalizations present. For hospitalizations that ended in intra-hospital death, YPLL were calculated as the difference between the age of the patient and the patient’s life expectancy based on their age and gender. The data source was SP.

### Statistical analysis

Both crude and standardized hospitalization rates were calculated. Age and sex-standardized hospitalization rates were calculated using the direct method, taking as a reference the 2015 European population prospect from the United Nations Population Division [26]. Descriptive statistics were used to present the distribution of hospitalizations for ACSCs by condition, age group, mean length of stay and the share of intra-hospital deaths.

For direct, lost productivity and total costs, cost per person and per hospitalization were calculated. For the direct and total costs per person, the population aged 18 or over of each country was the denominator. For the cost of lost productivity per person, the denominator was people aged 18 to 64 years, as people aged 65 years or older were considered not part of the workforce and therefore did not have associated lost productivity costs.

### Sensitivity analysis

Sensitivity analysis was done according to variations in the variables retirement age and age limit, presented as percent variation from base-case values. The retirement age used as the base-case was 65 years, for consistency and to facilitate comparison. Variations were made according to the respective official retirement ages for basic and minimum pensions in Portugal in 2015, which was 66 years.

Some other methodologies that identify hospitalizations for ACSCs limit the inclusion of older age groups [27, 28], as clinical complexity and increasing prevalence of comorbidities makes classifying hospitalizations as avoidable problematic for this population. Therefore, another variation in parameters for the sensitive analysis was to limit the definition of hospitalizations for ACSCs to those below 75 years. Both one-way (varying one parameter at a time) and multi-way (varying different parameters simultaneously) sensitivity analysis were performed. Costs per capita according to the variation on each parameter were also presented.

## Results

### Overview of hospitalizations for ACSCs

Table 1 shows an overview of hospitalizations in Portugal. A total of 99,417 hospitalizations were attributable to ACSCs, representing 10% of the total hospital admissions registered in 2015. Portugal presented crude and standardized rates of 1254 and 851 hospitalizations for ACSCs per 100,000 adults, respectively.

**Table 1:** Overview of population age distribution, overall hospitalizations and hospitalizations for ACSCs, Portugal, 2015

	Portugal
Total number of hospitalizations	1,000,670
Number of hospitalizations for ACSCs	99,417
Hospitalizations per ACSC^a^	
Bacterial pneumonia	35,523 (35.54%)
Congestive heart failure	22,753 (22.76%)
Hypertension	1896 (1.90%)
Chronic obstructive pulmonary disease (COPD) or asthma in older adults	10,470 (10.48%)
Asthma in younger adults	265 (0.27%)
Urinary tract infection	17,704 (17.71%)
Diabetes long-term complications	4541 (4.54%)
Diabetes short-term complications	1521 (1.52%)
Uncontrolled diabetes	993 (0.99%)
Lower-extremity amputation among diabetics	1266 (1.27%)
Dehydration	3019 (3.02%)
Hospitalizations for ACSCs per total of hospitalizations	9.93%
Rate of hospitalizations for ACSCs (per 100.000 population over 18 years old)	1253.88
Age and sex-standardized rate of hospitalizations for ACSCs (per 100.000 population over 18 years old)	850.60
Hospitalizations for ACSCs per sex	
Male	47,548 (47.83%)
Female	51,869 (52.17%)
Hospitalizations for ACSCs per age group	
18–44 years	4455 (4.49%)
45–64 years	13,573 (13.66%)
65+ years	81,389 (81.87%)
Mean age of patients hospitalized for ACSCs (standard deviation)	75.84 (14.54)
Mean length of stay in days for hospitalizations for ACSCs (standard deviation)	10.08 (11.22)
Hospitalizations for ACSC with death outcome (% of all hospitalizations for ACSCs)	13,453 (13.53%)
Per sex and age group	
Male	6732 (50.04%)
18–44 years	43 (0.32%)
45–64 years	492 (3.66%)
65+ years	6197 (46.06%)
Female	6721 (49.96%)
18–44 years	30 (0.22%)
45–64 years	194 (1.44%)
65+ years	6497 (48.29%)
Years of potential life lost	109,641

^a^ Some discharges due to lower extremity amputation also accounted for other diabetes-related ACSC

Hospitalizations for ACSCs were disproportionally concentrated in older-age groups (> 65 years), accounting for more than 80% of them. The mean age of patients hospitalized for ACSCs was 76 years (SD = 14.5) and the mean length of stay was 10 days (SD = 11.2). Women accounted for 52% of avoidable hospital admissions. Around 13.5% of the hospitalizations attributable to ACSCs ended with intra-hospital death. The most frequent cause of hospital admission was pneumonia (35.5%), followed by congestive heart failure (22.8%) and urinary tract infections (17.1%). Avoidable hospitalizations led to 109,641 YPLL.

### Costs related to hospitalizations

Table 2 presents the estimation of costs related to hospitalizations for ACSCs in Portugal. The total estimated cost associated with admissions for ACSCs was €250 million. Nearly 84% of this total cost comes from the direct cost of the hospitalization itself. Lost productivity costs are estimated to be around €40 million. Most of this value was due to premature death (€37.4 million). Absenteeism corresponded to 1% of the total estimated costs (€2.6 million). The total cost related to ACSCs corresponded to €31.53 per capita. This value represented the financial burden that was imposed on each adult inhabitant that could potentially have been avoided if ambulatory care was more effective.

**Table 2:** Estimated costs associated to hospitalizations for ACSCs, in €, 2015

	Total	Direct costs	Lost productivity	
Cost in €	250,064,177	210,026,755	40,037,422	
			*Absenteeism*	*Premature mortality*
			*2,631,311*	*37,406,111*
% of total cost		83.99%	16.01%	
Cost per person^a^	31.53	26.49	6.83	
Cost per hospitalization for ACSC^a^	2515.31	2112.58	2220.85	

^a^ For total and direct costs, the denominator was population over 18 years old. For lost productivity, the denominator was population between 18 and 64 years old

Table 3 details the cost of hospitalizations for each ACSC and by gender, and YPLL. Bacterial pneumonia accounted for a significant share of overall costs (€106 million; 41.9%), corresponding to a higher percentage than this condition represented in number of hospitalizations for ACSCs (Table 1). Bacterial pneumonia had a value of 63,905 YPLL, which is more than half of the YPLL for all ACSCs combined. Congestive heart failure and urinary tract infection were also important sources of costs associated with avoidable hospital admissions. The order in which these conditions account for total number of hospitalizations (Table 1) is the same as the order of cost distribution. Dehydration and bacterial pneumonia had the highest percentage of lost productivity representing total costs. These conditions presented the highest mortality rates among all ACSCs.

**Table 3:** Cost distribution and composition by ACSCs and sex, in €, 2015

ACSC	Total Costs (% all ACSCs costs)	Direct costs (% from total costs)	Lost productivity (% from total costs)	Mean total costs (95% CI)	Death rate	YPLL	Total costs Males (% all ACSCs costs)	Total costs Females (% all ACSCs costs)
Bacterial pneumonia	106,306,862 (41.90%)	80,546,077 (75.77%)	25,760,785 (24.23%)	2992 (2896; 3089)	21.20%	60,570	62,181,998 (24.51%)	44,124,864 (17.39%)
Congestive heart failure	59,334,934 (23.38%)	54,247,983 (91.43%)	5,086,951 (8.57%)	2608 (2545; 2670)	12.12%	21,539	27,690,862 (10.92%)	31,644,073 (12.47%)
Hypertension	3,109,537 (1.23%)	2,953,911 (95.00%)	155,626 (5.00%)	1640 (1507; 1773)	5.54%	726	1,571,535 (0.62%)	1,538,003 (0.61%)
COPD or asthma in older adults	22,618,306 (8.92%)	19,984,570 (88.36%)	2,633,735 (11.64%)	2160 (2067; 2254)	6.87%	6913	14,215,817 (5.6%)	8,402,488 (3.31%)
Asthma in younger adults	444,844 (0.18%)	422,311 (94.92%)	22,573 (5.08%)	1681 (1205; 2157)	0%	0	189,805 (0.07%)	255,667 (0.10%)
Urinary tract infection	29,616,268 (11.67%)	26,741,082 (90.29%)	2,875,186 (9.71%)	1673 (1623; 1722)	8.32%	11,785	11,887,808 (4.69%)	17,728,460 (6.99%)
Diabetes long-term complications	12,815,990 (5.05%)	11,598,869 (90.50%)	1,217,121 (9.50%)	2822 (2670; 2975)	4.54%	2241	7,941,403 (3.13%)	4,874,587 (1.92%)
Diabetes short-term complications	3,146,821 (1.24%)	2,700,532 (85.82%)	446,290 (14.18%)	2069 (1828; 2310)	7.76%	1212	1,405,176 (0.55%)	1,741,645 (0.69%)
Uncontrolled diabetes	1,352,185 (0.53%)	1304,232 (96.45%)	47,954 (3.55%)	1362 (1304; 1419)	2.52%	218	590,309 (0.23%)	761,876 (0.30%)
Lower-extremity amputation among diabetics	9,031,819 (3.56%)	8,329,937 (92.23%)	701,883 (7.77%)	7134 (6734; 7535)	12.16%	1707	5,643,650 (2.22%)	3,388,170 (1.34%)
Dehydration	5,913,323 (2.33%)	4,565,029 (77.20%)	1,348,294 (22.80%)	1959 (1722; 2196)	14.08%	3345	2,959,256 (1.17%)	2,954,067 (1.16%)
Acute ACSC	141,836,453 (56.72%)	111,852,188 (78.86%)	29,984,265 (21.14%)	2521 (2457; 2586)	16.76%	75,880	77,029,061 (30.80%)	64.807.392 (25.92%)
Chronic ACSC	108,227,724 (43.28%)	98,174,566 (90.71%)	10,053,157 (9.29%)	2509 (2464; 2554)	9.32%	33,761	56.941.238 (22.77%)	51,286,486 (20.51%)

### Sensitivity analysis

Table 4 presents the results of the sensitivity analysis. Extending the retirement age from 65 to 66 years led to an increase of 12% on costs associated with lost productivity in Portugal. The exclusion of people aged 75 years or older had a high impact on the estimation of direct costs, which reduced by 68%. The simultaneous variation in both parameters led to a 55% decrease in estimated total costs associated with hospitalizations for ACSCs. In this case, 39.6% of total costs were due to lost productivity.

**Table 4:** Sensitivity analysis results. Changes from Base-case, in %, according to different parameters on retirement age and age exclusions

	Direct cost	Lost productivity	Total
**Base-case (in €)**	**210,026,755**	**40,037,422**	**250,064,177**
Retirement age	0.00	+ 11.81	+ 1.89
Age limit			
Exclusion ≥75 years	−67.50	0.00	−56.69
Multi-way (retirement age and age limit)	−67.50	+ 11.81	−54.80
**Total costs per population aged 18 years or older (in €)**			
Base-case			31.53
Retirement age variation			32.13
Age limit variation			13.66
Multi-way variation			14.25

## Discussion

### Key findings

Hospitalizations for ACSCs involve high costs to both the health system and to individuals, and this situation is cause for concern, since these episodes are potentially avoidable. The cost of each avoidable hospitalization was estimated at €2515 for Portugal, indicating that substantial health resources could be saved by reducing the number of avoidable hospitalizations. In 2015, the total budget of public hospitals in Portugal was €4299 million [29]. Avoidable hospital admissions represented 6% of this value, indicating that they can be considered a major source of pressure on health system resources.

This study is the first to provide estimates of lost productivity costs associated with avoidable hospitalizations. Therefore, it is unfeasible to contrast the overall findings of this study with the existing literature. It is possible, however, to compare the direct costs estimated on previous studies with the ones found here. The previous study in Portugal that also used the AHRQ methodology found that, between 2000 and 2007, the mean yearly direct costs amounted to €200 million [11], which is similar to the €210 million estimated in this study. The previous studies in France and Ireland used DGRs. In France, they amounted to €5066 million in 2010 [8] (€3098 per hospitalization/ €80 per capita). In Ireland, costs were €352 million in 2008 [5] (€5055 per hospitalization/ €78 per capita).

It is important to emphasize that the choice of methodology used to select ACSCs codes leads to differences in the estimation of costs. The two studies in France and Ireland used different ACSC identification methodologies that included several conditions not considered in this study, such as vaccine-preventable conditions, angina, nutritional deficiencies and cellulitis, among others [5, 8]. The impact of differing definitions of ACSCs on quantitative results has been pointed out by Purdy et al. [6], in which total costs estimated in 2005 and 2006 in England could be between £1.183 and £1.714 billion, depending on the conditions considered. Furthermore, there are possible differences in the prevalence of diseases and the organization of the health care systems in Portugal, France and Ireland. In addition, there are substantial differences between DRGs systems in European countries [30].

The human capital approach employed in this study attributes higher values of lost productivity to younger people, due to the higher number of potentially productive years lost. Patients over the age of 65 accounted for 81% of the 99,000 hospitalizations for ACSC. Nonetheless, lost productivity due to absenteeism and premature death represented 16% of the total estimated cost. Studies produced in other countries analyzed lost productivity costs for some conditions (avoidable or not), including diabetes [31], asthma [32], heart failure [33] and COPD [34]. The proportion that lost productivity accounted for total costs in these studies ranged between 15 and 47% of total costs. The results show the importance of including lost productivity in cost estimation, to represent the economic impact more comprehensively.

When comparing frequencies and costs between genders, results show that although 52% of the avoidable hospitalizations occurred among women, they represented 46% of total costs. As each gender represented nearly half of all intra-hospital deaths, the difference can be seen both in the higher death rates among younger age groups for men, and their higher mean salaries and labor force participation, when compared to women. Therefore, lost productivity was the driver for men accounting for a higher share of total costs. Previous studies on costs for lost productivity for different conditions that analyzed gender differences also found higher costs for men [16–18].

The exclusion of people aged 75 years or older in the sensitivity analysis showed the relevance of age distributions to the cost composition. As direct costs account for a high share of total costs in Portugal, the estimation of total costs in this case became less than half of what was estimated on the base-case (from €250 million to €68 million). Concerning the variations in the retirement age, it is important to note that the average effective age of retirement in different countries varies from what is defined as official retirement age. In Portugal, there is the possibility of retiring early or continuing to work after retirement age. In fact, data from 2014 shows that, among the population between 65 and 69 years in Portugal, around 20% were still working [35].

Concerning cost distribution by condition, acute conditions were responsible for more than half of all avoidable hospital admissions and associated costs. Previous studies showed that distribution between avoidable conditions varied substantially between countries, with different conditions accounting for the largest proportion of total costs [5–8].

### Strengths and limitations

To our knowledge, this is the first study to estimate both direct and lost productivity costs associated with avoidable hospital admissions. Its strengths include the use of population data and a transparent and reproducible methodology. However, there are limitations in this study according to the method applied to estimate costs. The direct costs were estimated according to prices defined with the purpose of reimbursing hospitals (and therefore only cover public hospitals). These values do not reflect actual costs of an inpatient admission. Official prices are good proxy for real costs, are useful for health managers as they represent values reimbursed, allow the use of the same values for patients treated in different hospitals but with the same disease and have been used in other studies of cost estimation of avoidable hospitalizations [5, 6, 36].

When estimating lost productivity costs, the human capital measures potential lost productivity, instead of actual losses. The costs associated with premature death are not limited to the year of 2015, but also include the future lost potential such events represent. This methodology does not take into consideration the time it would require to replace a worker, how long it takes for the patient to return to work after discharge and the possibility of the patient not returning to the labor market due to being declared permanently incapable of work. There was no available information to calculate individually the patients experience after the discharge, as well as no indication if this population behaves the same as the average of hospitalized individuals for all causes. Costs were estimated using a standardized 30-day month; different approaches of estimation for daily wages provides different results; to consider only the working days in a month would require detailed information about the work of the patient, which were not available. Furthermore, the estimation for premature death does not take into consideration whether the death of the patient occurred after leaving the hospital, as it could still be associated with an ACSC. Therefore, the criteria selected might lead to underestimation of productivity loss.

There are other methods to estimate productivity loss costs, such as the friction cost method. The costs estimated by this method are expected to be lower, as it depends on the time organizations need to restore the initial production level in the absence of a worker [37]. Estimating this time span requires lots of information on labor market conditions that was not available [38]. The results may also change for a single country over time, depending on the macroeconomic context [38]. The human capital approach to the estimation is grounded in economic theory and is commonly used in the literature of cost estimation [15, 16, 38], enhancing comparability of results.

## Conclusions

The age distribution of avoidable hospitalizations had a significant impact on cost components in Portugal. One of the main findings of this study was that, although these hospital admissions had a substantial direct economic impact, they also imposed a considerable economic burden on society. Despite the methodological limitations on the estimation of costs, results indicate that substantial financial resources and YPLL could potentially be saved if the country reduced hospitalizations for ACSCs. Effective primary health care in the dimensions of accessibility, prevention and promotion are important to achieve such reduction. Reducing the number of avoidable hospitalizations can contribute to reduced hospital care use and alleviate healthcare-related financial pressures both on the state and on society, with positive results for the population.

## Data Availability

The data on all in-patient stays at all public non-specialized Portuguese NHS hospitals used in this study are available from Central Administration of the Health System, but restrictions apply to the availability of these data, which were used under license for the current study, and so are not publicly available. Data are however available from the authors upon reasonable request and with permission of the Central Administration of the Health System. The data on monthly wage, unemployment and labor force participation are available in the Statistics Portugal repository: https://www.ine.pt/xportal/xmain?xpid=INE&xpgid=ine_base_dados&xlang=en

## Abbreviations

ACSCs: Ambulatory Care Sensitive Conditions
AHRQ: Agency for Healthcare Research and Quality
COPD: Chronic obstructive pulmonary disease
DRGs: Diagnosis Related Groups
ICD: International Classification of Diseases
SP: Statistics Portugal
YPLL: Years of potential life lost
